# The Impact of Prepartum Depression and Birth Experience on Postpartum Mother-Infant Bonding: A Longitudinal Path Analysis

**DOI:** 10.3389/fpsyt.2022.815822

**Published:** 2022-05-30

**Authors:** Pia Eitenmüller, Siegmund Köhler, Oliver Hirsch, Hanna Christiansen

**Affiliations:** ^1^Department of Clinical Child and Adolescent Psychology, Philipps University Marburg, Marburg, Germany; ^2^Department of Obstetrics and Gynecology, University of Giessen-Marburg, Marburg, Germany; ^3^Department of Psychology, FOM University of Applied Sciences, Siegen, Germany

**Keywords:** pregnancy, prepartum depression, birth experience, primiparous and multiparous, postpartum mother-infant bonding

## Abstract

**Introduction:**

Negative effects of impaired postpartum mother-infant-bonding on mental health of mothers, their newborn children and subsequent child development are well documented. Previous research demonstrated an association between a negative birth experience and postpartum mental health affecting postpartum mother-infant bonding. This study investigates the extent to which prepartum depression and birth experience influence the postpartum mental health of mothers and their bonding toward their newborns, and whether these influences differ according to parity and self-reported prior mental health problems.

**Method:**

Three hundred and fifty-four women (18-43 years; *M* = 30.13, *SD* = 5.10) filled in the Edinburgh Postnatal Depression Scale (EPDS), the Maternal-Fetal Attachment Scale (MFAS), Salmon's Item List (SIL) assessing the birth experience, and the Postpartum Bonding Questionnaire (PBQ) at pre- and postpartum; they were also asked about birth complications and parity status.

**Results:**

Primipara reported significantly more birth complications (*p* = 0.048), with path analysis confirming this result (*p* < 0.001). Birth complications were associated with a more negative rating of the overall birth experience (*p* < 0.001). Mothers with self-reported prior mental health problems had higher prepartum depression scores (*p* < 0.001) but did not differ in other variables from mothers without prior self-reported mental health problems. Differences in depression scores between mothers with self-reported prior mental health problems and those without vanished at postpartum assessment (*p* > 0.05). Path-analysis highlighted the key role of postpartum depression, which was the only significant predictor of postpartum impairment in maternal-child bonding (*p* < 0.001). Birth experience and prepartum depression scores exerted an indirect effect on postpartum maternal-child bonding, mediated by postpartum depression.

**Discussion:**

The present study demonstrates the relevance of prepartum mental health of expectant mothers, especially of those who self-report prior mental health problems. The results support that reducing mental health problems of pregnant mothers might contribute to a more positive birth experience and potentially reduce postpartum depressive symptoms. As postpartum depression is associated with impaired parent-child bonding, such targeted interventions could promote child development. Group differences between primiparous and multiparous mothers suggest that the birth experience may be an influential factor for postpartum mental health.

## Introduction

Pregnancy and childbirth are life-changing events for parents. This time is associated with many positive feelings like anticipation and excitement, but also with negative ones like fear and uncertainty (1–3). In addition to physical changes and challenges (4–6), there are many psychosocial adjustments that need to be managed before, during, and after birth (7–9). In the following we address those time periods with the associated challenges as well as potential moderators and consequences for parent-child bonding.

### Prepartum

All events occurring between the first week of gestation until onset of childbirth are considered prepartum. Apart from the physical challenges that a pregnant woman may have to confront, e.g., water retention, (permanent) heartburn, impaired mobility, or gestational diabetes (5, 6), other psychosocial factors (e.g., concerns about partnership, financial situation, overall fear of what lies ahead) can lead to psychological stress associated with mental disorders, for instance depression (7, 10–13). There is evidence of depressive disorders beginning during pregnancy (14). Prepartum depression is classified under affective mental disorders in ICD-10 (15) and DSM-5 (16). With the accompanying symptoms of depressed mood, listlessness, loss of interest, diminished capacity for pleasure and enjoyment, anxiety symptoms, rumination, and emergent suicidal ideation, as well as psychomotor and cognitive impairments, prepartum depression does not differ much from other depressive disorders that can occur at any other time in life (15, 16). Prepartum depression is associated with negative effects on fetal development (17), and has been associated with prematurity, stillbirth, low birth weight, or with sudden infant death syndrome (18). Moreover, prepartum depressive symptoms, especially when they go untreated, raise the risk of birth complications, postpartum difficulties in parental mental health, and problems with parent-child bonding (19).

Apart from depressive symptoms, other mental stressors can influence the psychological well-being of mother and child. For example, prepartum anxiety is associated with obstetric interventions (20, 21) and can raise the risk of depression (22), or birth trauma (23). Moreover, prepartum bipolar symptoms appear to be associated with an accumulation of adverse pregnancy outcomes, such as hypertension and prepartum hemorrhage, and those affected by bipolar symptomatology also experienced more induced labor and Cesarean Sections [CS; (24)]. Regarding obsessive compulsive disorders (OCD), Williams and Koran (25), reported postpartum worsening of prepartum OCD symptomatology in 29% of their participating women. OCD symptomatology had not changed at all or for the better in up to 83% during pregnancy; 17% of women studied reported exacerbated symptoms during pregnancy, and 37% of these women later on reported postpartum depressive symptomatology.

### Birth

Birth refers to the delivery of the child and all factors that can occur during child birth. Risk factors negatively affecting the well-being and health of the mother and her newborn can also occur during childbirth. Birth complications are frequent and often unexpected (26, 27). Umbilical cord complications, placental dysfunction, positional anomalies, or labor disruption are among the most common complications of childbirth (28, 29). Obstetrical complications can increase the risk for acute stress reactions and postpartum depression (30), and they may also necessitate a CS. In Germany, the most common reasons for CS in 2013 were previous CS, worsening of the unborn baby's heartbeat, or the baby's breech presentation (31). Globally, CS rates have doubled over the past 15 years with large cross-cultural variability (32). Furthermore, findings on the effects of various obstetric interventions on the psychological well-being of parents and infants have been found. Sandall et al. (33) examined the short- and long-term effects of CS on the health of mothers and their children, and found that in comparison to children born vaginally, those born by CS are exposed to more short-term risks such as altered immune development, an increased likelihood of allergy, atopy, and asthma, and reduced intestinal gut microbiome diversity. They claim that this is because of the different hormonal, physical, bacterial, and medical confrontations infants are exposed to during birth. In addition, surgical procedures can trigger immediate health complications for the mother and complicate later pregnancies (33). Apart from such physical health consequences, there is evidence for negative psychological outcomes of a negative birth experience, such as an association with postpartum depressive symptoms (34), or the finding, that dissatisfaction with childbirth significantly reduced or delayed the desire for another pregnancy in mothers (35) and fathers (36). Furthermore, a traumatic birth experience (i.e., secondary CS) can result in fear and turbulent waves of panic, indicators of post-traumatic stress, during a subsequent pregnancy (37).

### Birth Moderators

Studies have shown that prepartum expectations are a significant moderator of birth and postpartum experiences (17, 38). Ayers and Pickering (38) for example postulated that expectations were related positively to birth experience. Bramadat and Driedger (39) reported a connection between unfulfilled birth expectations and satisfaction with labor, with more dissatisfaction in the case of unmet birth expectations. This finding has been replicated in other studies (40, 41). Fearing childbirth affects the experience of pregnancy and childbirth, as a pronounced fear of childbirth has been associated with a higher risk of depression (22), and of birth trauma (23). In addition to this, Ayers and Pickering (38) detected significant differences in expectations and experiences between primiparous and multiparous mothers. Primiparous women expected and experienced more negative emotions, more effective analgesia and better staff pain management; furthermore, they were more likely to judge giving birth as traumatic and challenging (38). Such findings are supported by a retrospective study showing that primiparous mothers revealed stronger discrepancies between expectations and experiences than multiparous ones (42). Primiparous required more obstetric interventions and had higher correlates with psychological variables, particularly regarding mother-infant bonding. However, retrospective measurements can be affected by recall-bias (43), though Jardine et al. (44) demonstrated in their English cohort that the rate of complicated births was higher for primiparous than multiparous mothers. Further studies investigated the extent to which expectation violations and negative birth experiences might have an unfavorable effect on parents and their newborn. Dorsch et al. (45) extracted expectations as a central link between prepartum childbirth expectations and postpartum well-being and concluded that the discrepancy between prepartum expectancies and birth experience could be decisive for whether and which postpartum psychological symptoms might arise. Furthermore, there is evidence for associations between prepartum expectations, depressive symptomatology and parent-child bonding (17).

### Postpartum

The term postpartum designates all events after childbirth. However, recovery from birth injuries or hormonal changes can take much longer (15, 16). In clinical practice and research, the definition of the postpartum period has been expanded to include each event up to 12 months after birth (14, 46, 47). Therefore, we will use this term when referring to events that can last or manifest up to 1 year after birth (48).

There is a relatively high risk for the development of mental health problems in the postpartum period (49). Postpartum depression (PPD), for example, is one of the most common mental disorders after childbirth (50). The 12-month prevalence of PPD varies widely with 0.5 % to 60 % by country and culture, with rates about 17% for Germany in 2006 (51). As most of the prevalence data do not provide incidence rates, Reck et al. (52) interviewed mothers 2 and 6 weeks after birth and determined a 3-month prevalence of 6.1% for PPD in Germany. The incidence was 4.6%, indicating that in 47 of the 1,024 participating mothers in their study, the depressive symptoms occurred for the first time after childbirth. As in prepartum depressive disorders, PPD symptomatology does not differ much from other depressive disorders that can occur at any other time of life, though those affected by PPD suffer more from spontaneous crying fits, reduced interest and sensitivity toward and acceptance of their infant, as well as sleep disturbances (53, 54). A PPD diagnosis should be given if criteria of depression are fulfilled and last longer than 14 days postpartum, as during the first 14 days feelings of sadness are experienced by up to 80% of mothers and this sadness usually vanishes after those 2 weeks (15, 16). Beyond the negative consequences on the parent's mental health, PPD symptoms affect the child's development including behavioral and attachment problems and impair emotional and cognitive development (55–58).

### Parent-Child Bonding

There are two terms for describing the relation between a mother and her child: *Attachment*, which is more relational and describing two-ways (from child's perspective toward the mother and vice versa) and *bonding*, which describes only one direction, e.g., in our study the sensations and feelings of a mother toward her child—and not the other way around. Throughout the remainder of the article we will use the term bonding for those feelings from mother to child. Bonding between parent and child begins prepartum (59) and even at this early stage plays a formative role for child development. Stronger prepartum parental bonding is associated with stronger postpartum bonding (60), while impaired parental bonding affects hormones, epigenetics, and the child's neural development (61). A strong mother-child bonding is associated with better maternal well-being (17) and positive prepartum and postpartum child development (61–66). As they develop, children securely attached to their parents seem to exhibit more social skills and have less difficulty with peers later on (67). Studies show that early secure bonding in social relationships results in fewer internalizing and externalizing problems, and greater social competence later on (65). These children also perform better cognitively (66), and seem to be significantly less affected by mental disorders in childhood, adolescence, and adulthood (63, 68). These findings highlight the sheer importance of secure bonding for the development and mental health of children.

There is ample evidence of the formative influence of a mother's mental disorder on the postpartum bonding quality between mother and child (69–74) with studies demonstrating a link between pre- and post-partum depression and bonding (56, 75). Reck and colleagues (52) for instance described impaired bonding in 7.1% of mothers with depressive symptoms 2 weeks after childbirth, and a more recent study even identified a 10% bonding impairment rate among all postpartum women hospitalized for depression (76). On the other hand, there are investigations failing to show such associations (77, 78). Those heterogeneous findings are reason enough to explore this link further, and to include factors such as birth complications and birth experience into our analyses, as they are known to influence maternal mental health.

### The Current Study

Summing up, giving birth to a child has been associated with mental health risks for mothers and this risk might be exacerbated in the case of prior mental health problems. Dayan et al. (11) demonstrated a significant correlation between prior mental health problems and prepartum depressive symptoms. As shown, prepartum mental disorders are associated with negative consequences on the birth experience and postpartum period (24, 25, 79). Research evidence also suggests a difference between primi- and multiparous mothers (38, 42). These birth experiences in turn seem to affect mothers' postpartum mental health, for example by promoting depressive symptoms (23, 34, 37). Postpartum depression in turn has been associated with an interference of mother-infant bonding (52, 56, 75, 76). Based on the research findings described above, we thus assume that mothers' prepartum mental health, birth experience and postpartum mental health affect postpartum mother-infant bonding (80). We hypothesized that:

1. Mothers with self-reported mental health problems prior to pregnancy differ from mothers without such problems in mental health pre- and post-partum;
2. Primiparous mothers differ from multiparous ones with respect to birth experience and complications;
3. Prepartum depressive symptoms are associated with a less positive birth experience;
4. Prepartum depressive symptoms and birth experience independently affect postpartum depressive symptoms;
5. Postpartum depression score (moderated by prepartum depressive symptomatology and negative birth experience) will be the main predictor for impaired postpartum maternal-child bonding.

## Methods

### Procedure

#### Recruitment

Recruitment took place via gynecological practices, the University Hospital Giessen-Marburg (UKGM), various midwives and the birth center Marburg. Study information and questionnaires were handed out by the first author in a one-on-one interview or in a group setting (e.g., as part of a childbirth preparation course). In addition, flyers with information about the online survey were distributed in various facilities such as daycare centers and pediatricians' offices. With the onset of the COVID-19 pandemic, the survey was mainly conducted online and disseminated on various social media internet sites.

#### Design

Our study was a longitudinal analysis with two assessments. Data collection was conducted from November 2019 until February 2021. Participants were asked to complete a self-assessment questionnaire before and within 6 months after childbirth. At the beginning of the study, expectant mothers were approached in person to complete a paper-pencil version of the questionnaire battery. With the onset of the COVID-19 pandemic, an online survey was launched to enlarge our sample via dissemination on social media networks. Apart from a current pregnancy there were no specific inclusion criteria for participation. In addition to the questionnaire, other ethically relevant documents were presented to the participants. Furthermore, participants had the option to provide an e-mail address if they wanted to receive a reminder about filling out the postpartum questionnaire.

#### Ethical Aspects

The study was approved by the Local Ethics Committee of the Department of Psychology at Philipps University Marburg (UMR) under the file number 2019-18k. All participants were informed about the general conditions regarding data protection, anonymity, and prerequisites. All participants received written study information and provided written informed consent to study participation. In order to be allowed to participate, a declaration of consent was signed by all participants or accepted via the online portal by “clicking” on a mandatory field. The participants were informed about their right to withdraw and about the procedure for deleting the data. A transparent explanation of data handling and participation revoke was ensured, and the voluntary nature of participation was highlighted. Anonymization was achieved with a personal code that participants assigned themselves. All data collected were kept confidential. Attention was drawn to the confidentiality of all project participants, and e-mail addresses. Expected birth dates were collected and kept separate from questionnaire data. It was guaranteed that no retroactive assignment of the data to the corresponding participants could take place.

### Measures

The data was collected via two self-assessment questionnaires (pre- and post-childbirth). In the first part of both questionnaires, participants completed a brief survey on general demographics such as age, educational attainment, number of children, and further self-reported whether they had experienced a prior mental disorder (“Have you been or are you currently receiving treatment for a mental disorder? If so, what is the assigned diagnosis”). The second part of the prepartum questionnaire contained questions regarding pregnancy and birth, such as experiences with abortions or stillbirths, gestation week, participation in a childbirth preparation course and complications during previous labors. The questionnaire administered postpartum contained questions about complications during the last childbirth (“Did you experience complications during your last birth?”) and difficulties in the postpartum period (“Are you having any problems in the postpartum period?”). The remaining parts of the survey were supplemented by standardized measurement questionnaires: The Edinburgh Postpartum Depression Scale (EPDS) was used both at pre- and post-partum to assess depressive symptomatology; the Maternal-Fetal-Attachment Scale (MFAS) was used for prepartum and the Postpartum-Bonding-Questionnaire (PBQ) for postpartum mother-child bonding; birth experience was assessed within the postpartum questionnaire using the Salmon's Item List (SIL). All standardized measures are explained in more detail below.

#### Edinburgh Postpartum Depression Scale

The Edinburgh Postpartum Depression Scale (EPDS) is a widely used self-report instrument assessing postpartum depression, consisting of 10 items on a four-point Likert scale (81). Due to its economic feasibility, it is readily used in clinics and research. It is only a screening instrument and cannot substitute for clinical examination. Cox et al. (81) determined a threshold score > 12 for major depression according to the DSM. Bergant et al. (82) translated the EPDS into German and based their validation on the ICD-10 criteria. Overall, a maximum sum score of 30 can be reached, which indicates severe depressive symptoms. An EPDS sum score of 0 indicates the absence of any depressive symptoms. Considering various degrees of severity, they determined an optimal threshold of 9.5, so that EPDS sum scores of at least 10 can already indicate a mild depressive episode with a sensitivity of 0.96, a specificity of 1, and a positive predictive value of 1 (82). With internal validity of *r* = 0.81 and internal consistency of α = 0.82, the German EPDS version demonstrates psychometric properties as good as the original version (83). Murray and Cox (84) validated the use of the EPDS in the prepartum period, whereupon Matthey et al. (85) conducted a stand-alone study to examine variability when applying cut-off values. They included the prepartum survey in their analyses and postulated the presence of a major depressive episode according to DSM-5 when an individual's prepartum EPDS score equaled ≥ 15 and the postpartum equaled ≥ 13. Since one of the purposes of the present study is to compare prepartum and postpartum depression scores, we considered the cut-off values proposed by Matthey et al. (85) for descriptive statistics. According to Muzik et al. (86), the postpartum survey's timing plays no significant role in diagnosing a PPD. Cronbach's alpha in our study achieved good reliability for prepartum EPDS1 (α = 0.80, CI of α [0.77, 0.83]) and postpartum EPDS2 (α = 0.84, CI of α [0.81, 0.86]).

#### Maternal-Fetal-Attachment-Scale

The Maternal-Fetal Attachment Scale (MFAS) is a self-assessment questionnaire assessing prepartum mother-fetus bonding (59). It was developed by Cranley in 1981 and revised by van den Bergh in 1989 (87). Dubber et al. (88) translated and validated Van den Bergh's revised version for German-speaking countries. The scale is composed of 24 items answered on a seven-point Likert scale. The sum score can range from 24 to 168. High scores indicate a stronger positive bonding to the fetus. Validation studies have revealed similar results, reporting a Cronbach's alpha for the total scale between α = 0.72 and α = 0.92 (89). The German version of the MFAS reveals at least satisfactory internal consistency (*ICC* = 0.77, Cronbach's alpha = 0.81) for the total scale (90). The MFAS proved to be adequately reliable across our sample (α = 0.79, CI of α [0.75, 0.82]).

#### Salmon‘s Item List

The Salmon's Item List (SIL) is a bipolar adjective scale assessing subjective birth experience on three subscales: “physical discomfort” “emotional distress” and “fulfillment” (91). For this study, we used the short 12-item version (92) of the validated German translation (93). The sum score ranges from 0 to 72, with a high SIL score indicating a positive birth experience and a low score a negative one (94). We recommend interpreting the total scale when using the short version (95, 96). There are various recommended cut-off values reflecting positive to negative birth experiences (92, 97). Since the method published by Alder et al. (97) for calculating the sum score allows an interpretation even if individual items were not answered, we relied on their proposed cut-off value of 36. The short version was validated based on a correlation test with the 20-item version that resulted in satisfactory internal consistency of α = 0.88 (92). The Cronbach's alpha for the 12-item version of the SIL achieved good reliability in our study (α = 0.86, CI of α [0.81, 0.90]).

#### Postpartum Bonding Questionnaire

The Postpartum Bonding Questionnaire (PBQ) is a self-report screening instrument for the early assessment of maternal postpartum bonding problems and has demonstrated satisfactory interrater reliability (75). Reck et al. (98) translated the PBQ into German and tested it on a German-speaking sample. A short version with 16 items entails the general factor “bonding impairment” that explains 23.9% of the overall variance of the scale with an internal consistency of α = 0.85. Each question is rated on a six-point Likert scale from 0 for “always” to 5 for “never.” The sum score ranges from 0 to 80 with a high PBQ Score associated with major bonding impairments. Eickhorst et al. (99) considered a cut-off score of 12 as high for the German 16-item version for both parents, thus indicating an impaired parent-child bonding. The PBQ's Cronbach's alpha achieved good reliability across our sample (α = 0.87, CI of α [0.80, 0.90]).

### Participants

A total of 354 pregnant women participated in our prepartum survey. The postpartum survey was completed by 131 mothers, resulting in a dropout rate of 62.0% (*N* = 223). The mean age prepartum was *M* = 30.13 years (*SD* = 5.10), postpartum *M* = 31.32 (*SD* = 4.86). The majority of participants had at least a high school diploma (55.6%). A total of 38.7% (*n* = 138) of the women claimed to be multiparous, whereas 53.4% (*n* = 189) reported being primiparous. Median gestation was 27 weeks (*M* = 26.11, *SD* = 9.33) at our prepartum survey. The pregnant mothers divided into *n* = 32 (9.0%) in the first (1st-12th week gestation), *n* = 163 (46.0%) in the second (13th-28th week gestation) and *n* = 154 (43.5%) in the third (29th-40th week gestation) trimester. In our postpartum sample of the survey (*N* = 131), the mean age of newborns was 8 weeks (*M* = 8.88 weeks, *SD* = 5.48). We formed three age groups for the newborns: 0-2 weeks *n* = 6 (4.6%), > 2 weeks <2 months [2nd-8th week of life: *n* = 88 (67.2%)], > 2 months and older [9th-24th week of life: *n* = 33 (25.2%)]. At the prepartum assessment, 15.3% of participating women reported suffering from prior mental health problems. At postpartum this proportional share rose by 3.8-19.1%. For detailed information on our sample's characteristics, see Supplementary Table 1.

### Statistical Methods

#### Group Comparisons

To examine differences between dropouts and completers, between primiparas and multiparas, and between mothers with and without self-reported prior mental health problems, we conducted a combination of ANOVAs and *t*-Tests (for continuous variables), and χ^2^-tests or Fisher's exact test (for categorical variables). In case of varied inhomogeneity, a Welch correction was executed. Reported *p*-values were corrected via Bonferroni-Holm adjustment. The groups were compared by applying these dependent variables: “prepartum depressive symptoms” (EPDS1), “postpartum depressive symptoms” (EPDS2), “birth experience” (SIL), “prepartum fetal bonding” (MFAS), “impairment in postpartum maternal-infant bonding” (PBQ), “birth complications” (birthcompl), and “difficulties postpartum” (DiffPuerp).

#### Sequential Longitudinal Pathway Analysis

**Figure 1:**
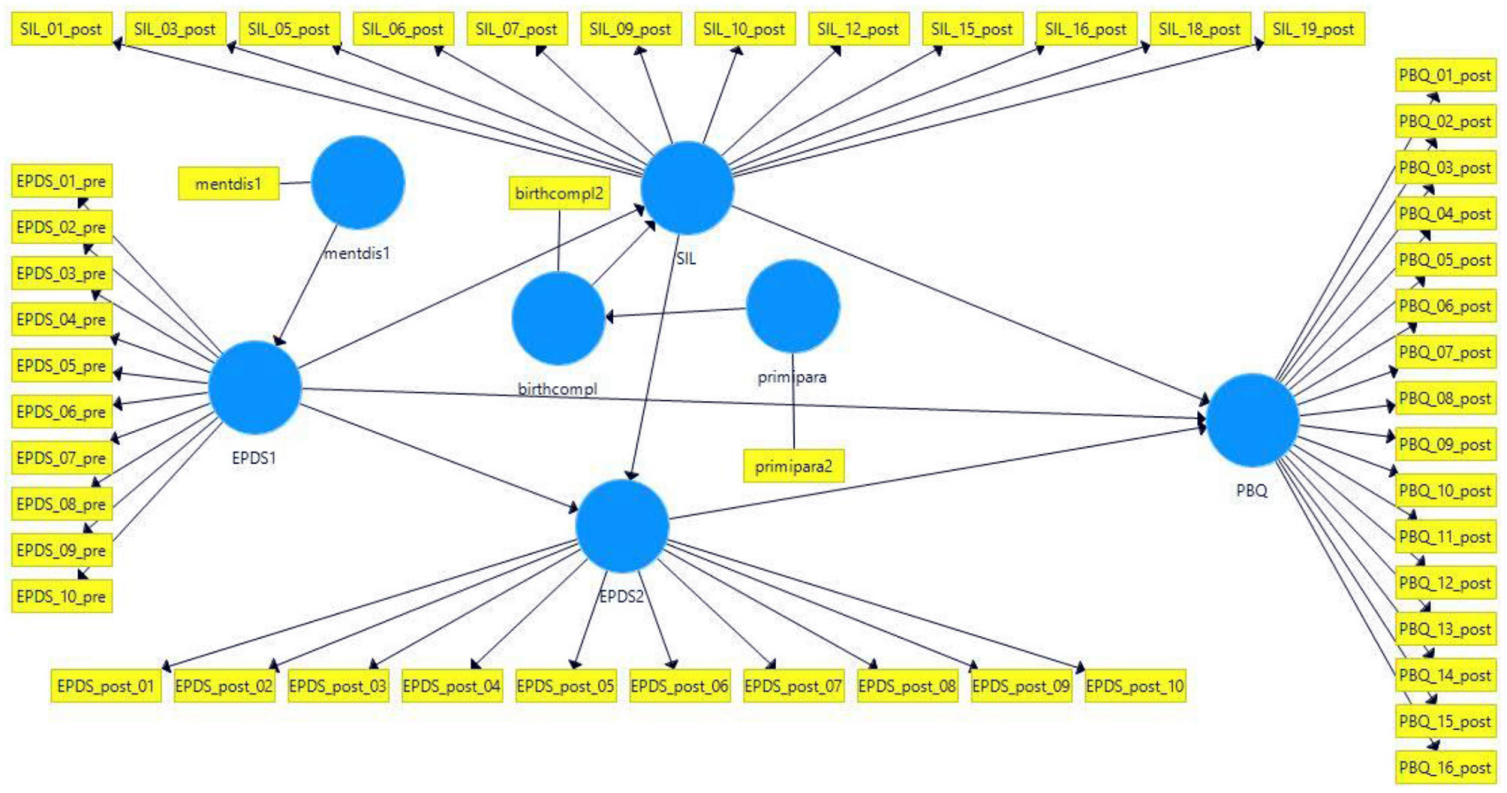
Partial Least Squares Structural Equation Model (PLS-SEM) predicting maternal bonding. mentdis1, Factor variable indicating self-reported prior mental health problems (prepartum); EPDS1, Prepartum depression score; birthcompl, Factor variable indicating birth complication at last labor (postpartum); SIL, Short version of Salmon's Item List to assess birth experience (postpartum); Primipara, Factor variable indicating primiparous women (prepartum); EPDS2, Postpartum depression score; PBQ, Postpartum impairment in mother-infant bonding.

Figure 1 shows the path model to be tested with all the paths derived theoretically between birth-related variables from hypothesis 1 to 4. The main predictor variables in the sequential model for the prediction of postpartum bonding (PBQ) are: EPDS scores pre- (EPDS1) and postpartum (EPDS2) as well as birth experience (SIL). Further, the effects of self-reported mental health problems (mentdis1) for EPDS scores prepartum, EPDS scores prepartum for EPDS postpartum, parity status (primipara) for birth complications and birth complications for birth experience (SIL) are tested sequentially for mediating effects. The model represents our assumptions of (1) the prediction of prepartum depression scores (EPDS1) *via* self-reported prior mental health problems (mentdis1); (2) the prediction of the birth experience (SIL) *via* reported birth complications (birthcompl), which in turn should be predicted by parity (primipara); and (3) the direct and indirect effects between the prepartum (EPDS1) and postpartum depression score (EPDS2), partially mediated by birth experience (SIL). Pathways from the birth experience (SIL) and prepartum depressive symptomatology (EPDS1) to postpartum bonding impairment (PBQ) are also mapped, which should not be significant according to our assumptions. Therefore, based on our theory, only the postpartum depression score (EPDS2) remains as a significant predictor of postpartum bonding impairment (PBQ).

We performed Partial Least Squares Structural Equation Modeling (PLS-SEM) predicting maternal bonding operationalized by the PBQ. PLS-SEM can be regarded as a variant of structural equation modeling which uses an ordinary least squares regression-based method (OLS) in contrast to the maximum likelihood estimation procedure in covariance-based structural equation modeling. PLS-SEM is a variance-based approach and can handle small sample sizes, complex models, and makes almost no assumptions about the level of measurement of data (100). To evaluate the measurement model, the outer (factor) loadings of variables on their respective latent constructs should be at least 0.708. On the other hand, variables with loadings ≥ 0.40 can also be included thanks to their contribution to content validity (101). Factor loadings can only be calculated if more than one manifest variable represents a latent construct. The internal consistency of latent constructs with more than one manifest variable is measured by the composite reliability. Values of 0.60 to 0.70 are acceptable in exploratory research (102). The average variance extracted (AVE) shows the proportion of variance the constructs explain in their indicators. It is equivalent to the communality in factor analysis and can be regarded as a measure of convergent validity. Discriminant validity is present if the indicators correlate highest with their constructs and do not have higher cross-loadings with other constructs. Furthermore, the heterotrait-monotrait ratio (HTMT) is calculated. This method is composed of the following two components: the correlations between the indicators that measure different constructs and correlations between indicators of the same construct. The value here should be below 0.85. Multicollinearity is present if indicators have a variance inflation factor (VIF) > 5 (100). The path weighting scheme was used for model estimation which standardizes the included variables. We bootstrapped with 5,000 samples to obtain tests of significance for path coefficients and outer loadings of variables forming latent constructs. The resulting *t*-values were then tested for significance. We considered a *p*-value of ≤ 0.05 to be significant. To evaluate the structural model, the coefficient of determination R^2^ can be used. Values of at least 0.75, 0.50, and 0.25 for endogenous latent variables are considered substantial, moderate, or weak, respectively (103). The effect size f^2^ demonstrates whether an exogenous construct has a substantive impact on an endogenous construct. Values of at least 0.02, 0.15, and 0.35 represent small, medium, and large effects, respectively (104). A limitation of this method is that there is no global goodness-of-fit criterion. We performed model calculations for subjects with complete data (*n* = 131) and subjects with missing data (*n* = 354) after imputation with the method of k nearest neighbor (kNN) using R package VIM (105). PLS-SEM calculations were done with the program SmartPLS 3 (106).

## Results

### Group Comparisons

Table 1 shows the means and standard deviations of all dependent interval scaled variables group-wise and across groups. High values on EPDS1, EPDS2, and PBQ indicate higher depressive symptomatology, respectively more strongly impaired mother-child bonding. In contrast, high scores on the MFAS and the SIL scales are associated with positive meanings. A high MFAS score reflects a strong and positive maternal-fetal bonding, and the higher the SIL score, the more positively the birth was experienced by participating mothers.

**Table 1:** Descriptive Data of interval scaled variables.

	**All participants *N* = 353**	**Dropouts** ***n* = 223**	**Completers *n* = 131**	**Primipara** ***n* = 189**	**Multipara *n* = 138**	**Self-reported mental health problems** ***n* = 54**	**No self-reported mental health problems *n* = 289**
**EPDS1**							
*M* *(SD)*	6.97 (3.86)	7.32 (3.97)	6.38 (3.60)	7.28 (3.95)	6.40 (3.41)	9.31 (4.70)	6.54 (3.54)
Test statistic		adj. *p* = 0.052, *d* = −0.25		adj. *p* = 0.150, *η^2^* = 0.01		adj. *p* < 0.001, *η^2^* = 0.21	
**MFAS**							
*M* (*SD*)	122.27 (19.01)	124.18 (18.72)	119.01 (19.14)	122.85 (17.82)	121.57 (17.95)	120.91 (16.92)	122.98 (18.07)
Test statistic		adj. *p* = 0.052, *d* = −0.27		adj. *p* = 0.522, *η^2^* < 0.01		adj. *p* > 0.05, *η^2^* < 0.01	
**SIL**							
*M* (*SD*)	51.34 (12.64)			49.70 (12.90)	54.83 (11.42)	45.29 (13.48)	52.73 (12.00)
Test statistic				adj. *p* = 0.150, *η^2^* = 0.04		adj. *p* = 0.06, *η^2^* = 0.05	
**EPDS2**							
*M* (*SD*)	6.46 (4.27)			6.97 (4.15)	5.38 (4.37)	7.10 (4.36)	6.31 (4.27)
Test statistic				adj. *p* = 0.150, *η^2^* = 0.03		adj. *p* > 0.05, *η^2^* < 0.01	
**PBQ**							
*M* (*SD*)	9.32 (6.77)			9.92 (7.10)	8.00 (5.85)	10.10 (7.20)	9.08 (6.67)
Test statistic				adj. *p* = 0.276, *η^2^* = 0.02		adj. *p* > 0.05, *η^2^* < 0.01	

*η^2^, eta square; d, Cohens d; adj. p, p-value after Bonferroni-Holm adjustment.*

*Group comparison with adjusted p-values and effect sizes of the ANOVAs and group-wise descriptive data of all dependent interval scaled variables. EPDS1, Prepartum depression score; MFAS, Maternal fetal attachment (prepartum); SIL, birth experience (postpartum); EPDS2, Postpartum depression score; PBQ, Postpartum impairment in mother-infant bonding*.

Table 1 also lists the adjusted *p*-values and effect sizes of the unifactorial analysis of variance (ANOVA), done to examine group differences between mothers with and without self-reported prior mental health problems and between primi- and multiparous mothers.

Table 2 shows the overall and group-wise distribution of dichotomous variables. Moreover, it indicates the cases of clinically relevant pre- and postpartum EPDS scores relying on the cut-off values by Matthey et al. (85). Note that the number of clinically relevant depression scores rose across all groups.

**Table 2:** Descriptive Data of categorically scaled variables.

	**Birth complications (birthcompl)**		**Postpartum difficulties (DiffPuerp)**		**Cases of clinically relevant EPDS scores**	
	**Yes**	**No**	**Yes**	**No**	**T1**	**T2**
All participants	57 (43.5 %)^b^	70 (53.4 %) ^b^	39 (29.8 %)^b^	91 (69.5 %)^b^	15 (4.2 %)^a, c^	16 (12.2 %)^b, c^
Primipara Multipara	46 (35.1 %)^b^ 11 (8.4 %)^b^	41 (31.3 %)^b^ 29 (22.1 %)^b^	31 (23.7 %)^b^ 8 (6.1 %)^b^	57 (43.5 %)^b^ 34 (26.0 %)^b^	8 (2.3 %)^a, c^ 2 (0.6 %)^a, c^	10 (7.6 %)^b, c^ 6 (4.6 %)^b, c^
Mothers with self-reported mental health problems Mothers without self-reported mental health problems	10 (7.6 %)^b^ 46 (35.1 %)^b^	10 (7.6 %)^b^ 60 (45.8 %)^b^	6 (4.6 %)^b^ 33 (25.2 %)^b^	14 (10.7 %)^b^ 76 (58.0 %)^b^	6 (1.7 %)^a, c^ 9 (2.5 %)^a, c^	4 (3.1 %)^b, c^ 12 (9.2 %)^b, c^

*Overall and group-wise distribution of dependent categorial scaled variables.*

*^a^Indicates the proportional number of total n = 354 mothers completing prepartum questionnaire.*

*^b^Indicates the proportional number of n = 131 mothers completing pre- and post-partum questionnaire.*

*^c^Cases of clinically relevant EPDS score identified by EPDS1 ≥ 15 and EPDS2 ≥ 13*.

#### Mothers With and Without Self-Reported Prior Mental Health Problems (Mentdis1)

Consistent with our expectations, mothers who self-reported prior mental health problems demonstrated significantly higher depression scores prepartum (*F*_*EPDS*1_ (1, 64.37) = 17.02, *p* < 0.001, adj. *p* < 0.001, η^2^ = 0.21, *CI* of η^2^[0.08, 0.35]). This effect disappeared postpartum (*F*_*EPDS*2_ (1, 128) = 0.59, *p* = 0.444, adj. *p* > 0.05, η^2^ <0.01, *CI* of η^2^[0.00, 0.04]). Descriptive examination of clinically relevant cases concerning depressive symptomatology, shown in Table 2, revealed an increase in relevant cases within the group of mothers with self-reported prior mental health problems from 1.7% at T1 to 3.1% at T2. The number of clinically relevant cases among previously self-reported healthy mothers rose from 2.5% at T1 to 9.2% at T2.

We detected no other significant effect in either the prepartum maternal-fetal bonding (*F*_MFAS_ (1, 76.58) = 0.67, *p* = 0.414, adj. *p* > 0.05, η^2^ <0.01, *CI* of η^2^[0.00, 0.07] nor postpartum in their subjective birth experience (*F*_*SIL*_ (1, 128) = 6.52, *p* = 0.012, adj. *p* = 0.06, η^2^ =.05, *CI* of η^2^[0.01, 0.12]), in postpartum bonding impairment (*F*_*PBQ*_ (1, 125) = 0.40, *p* = 0.529, adj. *p* > 0.05, η^2^ <0.01, *CI* of η^2^[0.00, 0.04]) or in the number of reported birth complications ($\chi_{\text{birthcompl}}^{2}$ = 0.30, *df* = 1, *p* = 0.586, adj. *p* > 0.05, *V* = 0.05, *CI* of *V*[0.00, 0.22]).

#### Primiparous vs. Multiparous (Primipara)

ANOVAs revealed no significant group differences between primipara and multipara in the continuous variable EPDS at T1 (*F*_*EPDS*1_ (1, 324) = 4.45, *p* = 0.036, adj. *p* = 0.150, η^2^ = 0.01, *CI* of η^2^[0.00, 0.04]), MFAS (*F*_MFAS_ (1, 325) = 0.41, *p* = 0.522, adj. *p* = 0.522, η^2^ <0.01, *CI* of η^2^[0.00, 0.02]) and SIL (*F*_*SIL*_(1, 129) = 4.85, *p* = 0.030, adj. *p* = 0.150, η^2^ = 0.04, *CI* of η^2^[0.00, 0.10]).

Neither were there any group differences in EPDS at T2 (*F*_*EPDS*2_ (1, 129) = 4.03, *p* = 0.047, adj. *p* = 0.150, η^2^ = 0.03, *CI* of η^2^[0.00, 0.09]) and PBQ (*F*_*PBQ*_ (1, 126) = 2.23, *p* = 0.138, adj. *p* = 0.276, η^2^ = 0.02, *CI* of η^2^[0.00, 0.07]), but primiparous were more likely to report birth complications than multiparous mothers ($\chi_{\text{birthcompl}}^{2}$ = 7.13, *df* = 1, *p* = 0.008, adj. *p* = 0.048, *V* = 0.24, *CI* of *V*[0.06, 0.41]).

#### Dropout Analyses

Our high dropout rate necessitated a dropout analysis. For this purpose, *N* = 131 complete data sets were compared with *N* = 223 dropout data in the variables of self-reported prior mental health problems, prepartum depression (EPDS1), age, number of children at the prepartum time, highest educational attainment, and prepartum maternal-fetal bonding (MFAS). Participants who failed to complete the second questionnaire were significantly younger at the prepartum time (*t*_*age*_ = 2.65, *df* = 282.21, *p* = 0.009, *adj. p* = 0.045, *d* =.30, *CI* of *d*[0.08, 0.52]), and had more children (*t*_*children*_ = −3.48, *df* = 322.73, *p* < 0.001, *adj. p* < 0.001, *d* = −0.38, *CI* of *d*[−0.60, −0.16]). After alpha adjustments, they did not differ in prepartum depression (*t*_*EPDS*1_ = −2.26, *df* = 294.47, *p* = 0.024, *adj. p* = 0.052, *d* = −0.25, *CI* of *d*[-0.46,−0.03]), maternal-fetal bonding (*t*_*MFAS*_ = −2.48, *df* = 267.73, *p* = 0.014, *adj. p* =0.052, *d* = −0.27, *CI* of *d*[-0.49,−0.06]), educational attainment ($\chi_{education}^{2}$ = 12.71, *df* = 4, *p* = 0.013, *adj. p* = 0.052, *V* = 0.19, *CI* of *V*[0.04, 0.28]) or distribution of mothers with vs. without self-reported prior mental health problems ($\chi_{\text{mentdis}1}^{2}$ = 0.10, *df* = 1, *p* = 0.746, *adj. p* = 0.746, *V* = 0.02, *CI* of *V*[0.00, 0.12]).

### Sequential Longitudinal Pathway Analysis

#### Complete Cases (n = 131)

Supplementary Table 2 displays the outer loadings of the multi-item scales in our original sample and after bootstrapping (sample mean) and their respective significance. Note that all factor loadings can be considered significant. Most do not reach the recommended cut-off value of 0.708 mentioned by Hair et al. (100), thus at least 50% of each indicator's variance is explained. On the other hand, the vast majority reached the criterion of ≥ 0.40 recommended by Brown (101). Only a few items (PBQ Items 4 and 7, EPDS1-items 2 and 10, SIL Item 16, and EPDS2 Item 10) have loadings <0.40.

Cronbach's alpha and composite reliability are used as the lower and upper bounds to assess the internal consistency reliability of multi-item scales. Cronbach's alpha for EPDS1 was 0.797, its composite reliability was 0.844, for EPDS2 the values were 0.840 and 0.875, for SIL 0.858 and 0.888, and for the PBQ 0.869 and 0.888, respectively. All values can be classified as satisfactory.

To evaluate convergent validity, we examined average variance extracted (AVE). The value for EPDS1 was 0.364, meaning that on average, the construct explains 36.4% of the variance of its indicators, which amounts to a construct's communality. For EPDS2 the value was 0.419, for SIL it was 0.418, and for the PBQ it was 0.340. The threshold of 0.50 was not reached, which is not surprising as the AVE is calculated as the sum of the squared loadings divided by the number of indicators. As most of the loadings were below 0.708 (Supplementary Table 2), an AVE of 0.50 could not be reached but the majority of factor loadings is nevertheless acceptable (101).

All of the indicators' loadings on the associated variables have higher loadings than their correlations with the other constructs. Thus, our data meet the first criterion of discriminant validity. All HTMT ratios were well beyond 0.85 and therefore discriminant validity was met.

### Structural Model

We examined multicollinearity by relying on the variance inflation factor (VIF). As just two SIL items slightly exceeded the threshold of 5, relevant multicollinearity cannot be assumed (107).

Table 3 displays standardized path coefficients between the latent constructs in the PLS-SEM model. Path coefficients should be at least 0.20, whereby values of > 0.40 can be interpreted as high if the model is complex. According to Hair et al. (100), a model is complex if there are four or more constructs, as is the case here. As seen in Table 3, there is a relevant association between EPDS2 and PBQ. However, the EPDS1 also yielded a significant result—contrary to our hypothesis.

**Table 3:** Structural model with complete cases (*n* = 131).

**Variables**	**Original sample (O)**	**Sample mean** ** (M)**	**Standard deviation (STDEV)**	**T statistics** ** (|O/STDEV|)**	***P*-value**
EPDS1 -> EPDS2	0.283	0.293	0.095	2.974	0.003
EPDS1 -> PBQ	0.179	0.178	0.087	2.058	0.040
EPDS1 -> SIL	−0.301	−0.326	0.092	3.288	0.001
EPDS2 -> PBQ	0.444	0.460	0.084	5.289	<0.001
birthcompl -> SIL	−0.332	−0.333	0.069	4.814	<0.001
mentdis1 -> SIL	0.312	0.321	0.093	3.367	0.001
SIL -> EPDS2	−0.392	−0.393	0.101	3.871	<0.001
SIL -> PBQ	−0.135	−0.136	0.085	1.585	0.113
Primipara -> birthcompl	0.217	0.219	0.083	2.611	0.009

*Standardized path coefficients between latent constructs*.

The R^2^ adjusted value after bootstrapping for maternal bonding (PBQ) was 0.408 and thus tended to move in a moderate direction.

A medium f^2^ effect size of 0.258 after bootstrapping showed a substantive impact of EPDS2 on maternal bonding (PBQ). The impacts of EPDS1 (0.054) and SIL (0.033) on maternal bonding (PBQ) were small, while another substantive impact of SIL on EPDS2 (0.226) was evident.

#### Imputation of Missing Data (n = 354)

The same model displayed in Figure 1 was tested in a sample of mothers whose missing values were imputed by the k nearest neighbor method (105).

Supplementary Table 3 displays the outer loadings of the multi-item scales in the original sample and after bootstrapping (sample mean) and their respective significance. Note that all factor loadings can be considered significant. As most did not reach the recommended cut-off value of 0.708 mentioned by Hair et al. (100), at least 50% of each indicator's variance is explained. On the other hand, the majority reached the criterion of ≥ 0.40 recommended by Brown (101). A few items (PBQ Items 4, 7, and 13, EPDS1 Item 10, SIL Item 1, 3, 5, 7, and 18, and EPDS2 Item 10) have loadings <0.40.

We used Cronbach's alpha and composite reliability as the lower and upper bounds to assess the internal consistency reliability of multi-item scales. Cronbach's alpha for EPDS1 was 0.804, composite reliability was 0.851, for EPDS2 the values were 0.819 and 0.861, for SIL post 0.796 and 0.805, and for the PBQ 0.848 and 0.871, respectively. All values can be classified as satisfactory.

We again examined AVE to assess convergent validity. The EPDS1 value was 0.373, meaning that on average, the construct explains 37.3% of its indicators' variance. The value was 0.402 for EPDS2, 0.295 for SIL post, and 0.312 for the PBQ. The threshold of 0.50 was not reached, but most factor loadings are nevertheless acceptable (101).

All of the indicators' loadings on the associated variables have higher loadings than their correlations with the other constructs; our data thus meet the first criterion of discriminant validity. All HTMT ratios were well beyond 0.85, thereby fulfilling discriminant validity

Table 4 displays standardized path coefficients between the latent constructs in the PLS-SEM model. The results in Table 4 confirm the unique influence of EPDS2 on PBQ. Neither the birth experience (SIL) nor prepartum depression score exerted a direct influence on PBQ. Furthermore, our results support the hypothesis that prepartum depressive symptoms predicted a more negative evaluation of childbirth, thus moderately amplifying the EPDS1 effect on EPDS2. Additionally, our path analysis' output confirmed the findings from our group comparisons, meaning that EPDS1 was significantly predicted by self-reported prior mental health problems (mentdis1) and birth complications were predicted by parity (primipara reported more complications).

**Table 4:** Structural model with imputed missing data (*n* = 354).

**Variables**	**Original sample (O)**	**Sample mean** ** (M)**	**Standard deviation (STDEV)**	**T statistics** ** (|O/STDEV|)**	***P*-value**
EPDS1 -> EPDS2	0.267	0.268	0.047	5.680	<0.001
EPDS1 -> PBQ	0.064	0.061	0.048	1.337	0.181
EPDS1 -> SIL	−0.270	−0.277	0.054	5.020	<0.001
EPDS2 -> PBQ	0.642	0.651	0.046	14.086	<0.001
birthcompl -> SIL	−0.371	−0.371	0.044	8.437	<0.001
mentdis1 -> EPDS1	0.269	0.271	0.059	4.539	<0.001
SIL -> EPDS2	−0.408	−0.414	0.054	7.623	<0.001
SIL -> PBQ	−0.044	−0.041	0.057	0.771	0.441
Primipara -> birthcompl	0.260	0.260	0.050	5.205	<0.001

*Standardized path coefficients between latent constructs*.

The R^2^ adjusted value after bootstrapping for maternal bonding (PBQ) was 0.489 and thus moved in a moderate direction.

A medium f^2^ effect size of 0.579 after bootstrapping showed a strong EPDS2 impact on maternal bonding (PBQ). The impacts of EPDS1 (0.01) and SIL (0.007) on maternal bonding (PBQ) were negligible, while another substantive impact of SIL post on EPDS2 (0.234) was evident.

## Discussion

Our analyses support our first hypothesis, that mothers with a self-reported prior mental health problem differ significantly from mothers without such prior problems. Mothers with prior mental health problems reported more depressive symptoms according to EPDS scores, in line with other findings of the field (11). However, at postpartum assessment this difference vanished. In light of this, the different cut-off values at pre- and post-partum should be considered as suggested by Matthey et al. (85). They conducted a study to validate prepartum and postpartum cut-off scores and recommend a higher cut-off score > 15 at pre- and > 13 at post-partum to indicate clinically relevant depression symptoms. Otherwise, applying an unvalidated cut-off value at prepartum can result in an almost three-fold overestimation of depression rates (85). In our study, descriptive data allowed us to determine that the proportional share of clinically relevant depression scores rose across all groups. At postpartum, the proportion of clinically relevant depression scores almost doubled among mothers with self-reported prior mental health problems, but more than tripled in the group of mothers who did not report such prior problems, indicating a convergence of groups with respect to depressive symptoms. This alignment may have resulted from different factors: first, childbirth can be a particularly overwhelming and sometimes even traumatic event for parents (23), e.g., due to the risks of unexpected birth complications and related negative effects on maternal mental health (26, 27, 33, 49). Further, the postpartum period is a high-risk time for developing mental health problems (49), especially with regard to depression (50–52). The 4.6% incidence rate of PPD determined by Reck et al. (52) reflects the proportion of mothers who did not report any mental health problems at prepartum, but developed clinically relevant depressive symptoms at postpartum.

Regarding our second hypothesis, we were able to demonstrate significant differences between primiparous and multiparous mothers. Primipara reported more birth complications (42, 44) which in turn contributed to a less positive birth experience (30, 38), overall replicating results of the literature. This calls for further research on the question, how prepartum knowledge on birth could contribute to a more informed expectation of this experience in primipara, that in turn could reduce anxiety and encourage more realistic expectations about birth, as anxiety and unfulfilled expectations seem to be associated with more obstetric interventions and a negative birth experience (20, 23, 108). There seem to be significant differences in the expectations of multipara (38), suggesting that prior experiences and informed expectations vs. a discrepancy between (uninformed) expectations and the actual birth experience in primipara may be a formative factor for developing mental health problems (45). However, primiparous women did not differ significantly from multiparous ones in pre- and post-partum depression scores or maternal-fetus bonding. Path analysis revealed an effect of prepartum depressive symptoms on birth experience (19, 79), though, and this had an effect on EPDS scores at postpartum (18, 19), thus confirming our third hypothesis and similar results of the literature (34).

With respect to our fourth hypothesis, our results demonstrate that birth experience is influenced by prepartum depression scores as well as by birth complications (especially in primipara). Prepartum depression scores and birth experience had an independent effect on the postpartum depression scores and those proved to be the main predictor for impaired postpartum maternal-child bonding, moderated by prepartum depressive symptoms and birth experience, thus proving our final hypothesis. The results of our pathway analysis confirm the influence of postpartum depression symptomatology on postpartum bonding shown in prior research (52, 56, 72, 75, 76). However, in our study, neither birth experience nor the prepartum depression score were directly associated with postpartum mother-infant bonding. This finding highlights the unique role that postpartum depressive symptomatology plays in impairing the postpartum bond. With regard to the negative long-term influences of such an impaired mother-child bonding on a child's development (61), e.g., reduced social and cognitive competences (65, 66), difficulties with peers (67), a higher risk for internalizing and externalizing problems (65) and mental disorders (63, 68), this highlights the need to closely monitor depressive symptoms and psychological distress during pregnancy and postpartum, and to improve pregnancy care as to prevent postpartum bonding problems and later child development problems (109–111). Of note, all postpartum measures were assessed at the same time. It is thus not clear whether postpartum depression influences bonding or the other way around, although there is more support in the scientific literature of the first assumption. However, cross-lagged panel studies with more assessments postpartum are needed for clarification.

Overall, our findings demonstrate how different pregnancy- and birth-related variables can influence each other and affect the mental health of mothers and subsequently their child bonding. It is therefore essential that we continue to improve our understanding of how exactly pre- and post-partum mental health as well as the birth experience affects the mental health of mothers and their newborn. In particular, the potential association between a negative birth experience and postpartum posttraumatic stress disorder (23, 112, 113) should be further explored in the context of potential postpartum bonding difficulties (114). However, the majority of participants in our study did have scores within the normal range, that is only a small proportion of mothers reported EPDS values above a critical cut-off and the same holds true for bonding. Future studies should aim at identifying impaired mothers/fathers and assess their difficulties in comparison to parents without such impairments.

### Limitations

There are several limitations to be considered. First, we were not able to continue recruiting in person due to the onset of the COVID-19 pandemic and had to resort to a digital questionnaire, which made it almost impossible to foster feelings of commitment and interest of the participants regarding our post-survey. The pandemic has caused additional psychosocial stress in almost all areas of life and for some time partners were not allowed to be present at birth. Those multidimensional stressors (115) could have independently contributed to mental health impairments of expecting as well as new mothers. Furthermore, it may explain our high drop-out rate, as families were in a sort of state of emergency at this time and had many new challenges to deal with.

Second, drop-out rates were large. Analysis indicated that mothers who dropped out were younger and had more children, though they did not differ from completers with respect to prepartum depression scores, self-reported mental health problem, maternal-fetal bonding and educational attainment. Thus, the analyses with data imputation are not biased in that respect. However, having more children does require more capacities from parents and it might be that families with younger mothers are still in a phase of consolidating family and work which is highly demanding. As reminder letters were a voluntary option, mothers may not have remembered the survey after the birth of their child. Or else, those mothers who did remember may have been particularly healthy and not as stressed in the postpartum period, which could represent a further bias in the sample. Therefore, to compensate for missing post-measurements, we conducted thorough statistical analyses on the complete pre- and post-data set and replicated results with imputations with the method of k-nearest neighbor, that resulted in similar findings.

Third, previous research found differences in depression symptoms and fear of childbirth over the course of pregnancy. While we had mothers in all stages of pregnancy (1st to 3rd trimester) this unstandardized assessment might have influenced results.

Fourth, mental health problems were only assessed based on self-report with one question in the pre- and post-partum survey. Additionally, the number of participants reporting (prior) treatment of a mental disorder was small. Those questions were dummy coded (yes/no) for analyses. There is the possibility of mothers not answering truthfully, although we assume that mothers who do report on prior mental health problems and birth complications did in fact experience those. Still, those aspects limit the validity and clinical implications of the result. In follow-up studies, participants should undergo a comprehensive clinical examination in addition to self-report questionnaires to obtain more valid information about the mental health and potential diagnosis.

Fifth, we did neither assess whether pregnancies were planned nor cultural background. An unplanned pregnancy might be a pleasant surprise or a significant stressor, and factors such as cultural background affect this. Such stress factors should thus be addressed in the future.

Sixth, we did not assess physical complaints/symptoms during or after pregnancy. As outlined in the introduction, such physical complaints (5, 6) have been associated with psychological distress and should be considered in future work.

Last, though we aimed at including partners, the sample of participating partners was so small that it was not possible to include them in analyses. Partners play a crucial role during pregnancy and in the postpartum period as depression scores of mothers and their partners correlate significantly and are predicted by perceived parenting stress (116). While there is research examining the impact of pregnancy and childbirth on the mental health of partners (7, 72, 79, 96, 114), there are few studies addressing the effects of birth complications on partners (117). Future studies should thus aim at including mothers and fathers in such studies.

### Conclusion and Outlook

This study demonstrates that mother-child bonding is significantly influenced by maternal postpartum depressive symptoms, and those are predicted by prepartum depressive symptoms as well as the birth experience. Accordingly, pregnant females should undergo regular screening during pregnancy to facilitate early identification and treatment of mental health problems. Postpartum aftercare should be offered more frequently and continue until at least the infant's first birthday, focusing not only on the child's development, but also on the mother's psychological well-being, especially concerning postpartum depression symptomatology.

## Data Availability Statement

The raw data supporting the conclusions of this article will be made available by the authors, without undue reservation.

## Ethics Statement

The studies involving human participants were reviewed and approved by Department of Psychology at Philipps-University Marburg (UMR) under file number 2019-18k. The patients/participants provided their written informed consent to participate in this study.

## Author Contributions

HC and PE contributed to conception and design of the study. SK supported site recruitment. PE organized study implementation and data collection and wrote the first draft of the manuscript. PE and OH performed statistical analysis. All authors contributed to manuscript revision, read, and approved the submitted version.

## Funding

Open Access funding provided by the Open Access Publication Fund of Philipps University Marburg with support of the Deutsche Forschungsgemeinschaft (DFG, German Research Foundation).

## Conflict of Interest

The authors declare that the research was conducted in the absence of any commercial or financial relationships that could be construed as a potential conflict of interest. Leah Millard, The University of Manchester, UK in collaboration with reviewer MWW.

## Publisher's Note

All claims expressed in this article are solely those of the authors and do not necessarily represent those of their affiliated organizations, or those of the publisher, the editors and the reviewers. Any product that may be evaluated in this article, or claim that may be made by its manufacturer, is not guaranteed or endorsed by the publisher.
