# Supplementary material for: The Impact of Prepartum Depression and Birth Experience on Postpartum Mother-Infant Bonding: A Longitudinal Path Analysis
Source: Front Psychiatry. 2022 May 30;13:815822. doi: 10.3389/fpsyt.2022.815822 (PMC9189288; doi:10.3389/fpsyt.2022.815822)
Supplement: Supplementary file 1 [file Table_1.pdf]

Supplementary Material

1 Sample Characteristics

Supplementary Table 1 Sample Characteristics.

All participants, n (%)	354 (100.0)
Demographic variables	
[b]Age mean in years, *M* (*SD*)	31.32 (4.86)
[a]Parity	
Primipara, n (%)	189 (53.4)
Multipara, n (%)	138 (39.0)
[a]Highest educational level	
Secondary school diploma, n (%)	39 (11.0)
Completed vocational training, n (%)	89 (25.1)
High school diploma, n (%)	33 (9.3)
Academic degree, n (%)	164 (46.3)
Other qualification, n (%)	28 (7.9)
Treatment of mental disorder	
[a]Mothers with self-reported (prior) treatment, n (%)	54 (15.3)
[a]Mothers without self-reported (prior) treatment, n (%)	298 (84.2)
[b]Mothers with self-reported (prior) treatment, n (%)	25 (19.1)
[b]Mothers without self-reported (prior) treatment, n (%)	106 (80.9)
Birth-related variables	
[a]Gestation week, *M* (*SD*)	26.11 (9.32)
[a]Pregnancy Trimester	
First trimester (1st-12th week gestation), n (%)	32 (9.0)
Second trimester (13th-28th week gestation), n (%)	163 (46.0)
Third Trimester (29th-40th week gestation), n (%)	154 (43.5)
Birth complications	
[ac]Previous experience with birth complications, n (%)	50 (14.1.)
[b]Complications during the last birth, n (%)	57 (43.5)
[b]No complications during the last birth, n (%)	70 (53.4)
[b]Age of the newborn in weeks, *M* (*SD*)	8.88 (5.48)
Under to 2nd week of life), n (%)	6 (4.6)
Under 2 months (2nd-8th week of life), n (%	88 (67.2)
Above 2 months (9th-24th week of life), n (%)	33 (25.2)
[bd]Difficulties Postpartum	
Difficulties postpartum, n (%)	39 (29.8)
No difficulties postpartum, n (%)	91 (69.5)

[a]at prenatal survey (with total *N* = 354)
[b]at postpartum survey (with total *N* = 131)
[c]Previous experience of birth complications includes a primipara's experience of abortion
[d]Dichotomous variable which could include any situation the participants experienced as stressful after birth
