# Supplementary material for: The Impact of Prepartum Depression and Birth Experience on Postpartum Mother-Infant Bonding: A Longitudinal Path Analysis
Source: Front Psychiatry. 2022 May 30;13:815822. doi: 10.3389/fpsyt.2022.815822 (PMC9189288; doi:10.3389/fpsyt.2022.815822)
Supplement: Supplementary file 2 [file Table_2.pdf]

2 Measurement model with complete cases (*n* = 131)

Supplementary Table 2 Measurement model with complete cases (*n* = 131).

Variables	Original Sample (O)	Sample Mean (M)	Standard Deviation (STDEV)	T Statistics (|O/STDEV|)	p Value
**PBQ**					
Item 1	0.770	0.760	0.057	13.486	<0.001
Item 2	0.570	0.555	0.099	5.762	<0.001
Item 3	0.650	0.639	0.086	7.579	<0.001
Item 4	0.353	0.362	0.144	2.443	0.015
Item 5	0.669	0.667	0.056	12.048	<0.001
Item 6	0.509	0.514	0.108	4.705	<0.001
Item 7	0.395	0.346	0.183	2.154	0.031
Item 8	0.529	0.511	0.116	4.562	<0.001
Item 9	0.632	0.630	0.081	7.802	<0.001
Item 10	0.682	0.680	0.075	9.143	<0.001
Item 11	0.580	0.578	0.093	6.252	<0.001
Item 12	0.632	0.626	0.067	9.376	<0.001
Item 13	0.587	0.569	0.094	6.264	<0.001
Item 14	0.418	0.421	0.081	5.129	<0.001
Item 15	0.710	0.707	0.058	12.139	<0.001
Item 16	0.451	0.452	0.096	4.707	<0.001
**EPDS1**					
Item 1	0.536	0.525	0.097	5.530	<0.001
Item 2	0.371	0.362	0.118	3.151	0.002
Item 3	0.528	0.525	0.082	6.410	<0.001
Item 4	0.550	0.542	0.084	6.569	<0.001
Item 5	0.718	0.712	0.062	11.669	<0.001
Item 6	0.664	0.659	0.059	11.197	<0.001
Item 7	0.640	0.632	0.071	9.074	<0.001
Item 8	0.793	0.788	0.040	20.005	<0.001
Item 9	0.733	0.727	0.061	12.083	<0.001
Item 10	0.327	0.321	0.091	3.590	<0.001
**SIL**					
Item 1	0.703	0.699	0.063	11.071	<0.001
Item 3	0.718	0.713	0.064	11.173	<0.001
Item 5	0.781	0.776	0.054	14.449	<0.001
Item 6	0.684	0.680	0.065	10.449	<0.001
Item 7	0.780	0.776	0.049	16.062	<0.001
Item 9	0.818	0.816	0.040	20.685	<0.001
Item 10	0.824	0.820	0.035	23.734	<0.001
Item 12	0.551	0.546	0.085	6.517	<0.001
Item 15	0.472	0.468	0.086	5.490	<0.001
Item 16	0.276	0.273	0.103	2.675	0.007
Item 18	0.508	0.498	0.096	5.314	<0.001
Item 19	0.335	0.334	0.098	3.425	0.001
**EPDS2**					
Item 1	0.728	0.729	0.047	15.440	<0.001
Item 2	0.566	0.569	0.067	8.485	<0.001
Item 3	0.774	0.772	0.035	22.314	<0.001
Item 4	0.645	0.642	0.054	12.011	<0.001
Item 5	0.711	0.709	0.058	12.183	<0.001
Item 6	0.510	0.506	0.085	6.033	<0.001
Item 7	0.571	0.564	0.083	6.903	<0.001
Item 8	0.727	0.724	0.047	15.463	<0.001
Item 9	0.744	0.745	0.048	15.538	<0.001
Item 10	0.390	0.394	0.101	3.848	<0.001

Supplementary Table 2 Outer (factor) loadings of variables on their latent constructs
