# Supplementary material for: The Impact of Prepartum Depression and Birth Experience on Postpartum Mother-Infant Bonding: A Longitudinal Path Analysis
Source: Front Psychiatry. 2022 May 30;13:815822. doi: 10.3389/fpsyt.2022.815822 (PMC9189288; doi:10.3389/fpsyt.2022.815822)
Supplement: Supplementary file 3 [file Table_3.pdf]

3 Measurement model imputing missing data (*n* = 354)

Supplementary Table 3 Measurement model imputing missing data (*n* = 354)

Variables	Original Sample (O)	Sample Mean (M)	Standard Deviation (STDEV)	T Statistics (|O/STDEV|)	*p* Value
**PBQ**					
Item 1	0.731	0.729	0.029	25.048	<0.001
Item 2	0.422	0.416	0.060	7.057	<0.001
Item 3	0.663	0.661	0.039	16.996	<0.001
Item 4	0.297	0.302	0.097	3.070	0.002
Item 5	0.727	0.727	0.031	23.141	<0.001
Item 6	0.573	0.576	0.070	8.163	<0.001
Item 7	0.231	0.211	0.104	2.224	0.026
Item 8	0.441	0.437	0.061	7.202	<0.001
Item 9	0.615	0.614	0.063	9.716	<0.001
Item 10	0.765	0.765	0.036	20.988	<0.001
Item 11	0.631	0.631	0.043	14.639	<0.001
Item 12	0.550	0.550	0.046	11.990	<0.001
Item 13	0.360	0.354	0.070	5.168	<0.001
Item 14	0.517	0.518	0.052	9.896	<0.001
Item 15	0.615	0.614	0.040	15.203	<0.001
Item 16	0.464	0.463	0.066	7.067	<0.001
**EPDS1**					
Item 1	0.614	0.612	0.043	14.202	<0.001
Item 2	0.458	0.454	0.060	7.662	<0.001
Item 3	0.529	0.527	0.049	10.823	<0.001
Item 4	0.540	0.538	0.051	10.691	<0.001
Item 5	0.721	0.719	0.035	20.366	<0.001
Item 6	0.649	0.647	0.036	18.171	<0.001
Item 7	0.611	0.609	0.046	13.304	<0.001
Item 8	0.783	0.780	0.025	31.410	<0.001
Item 9	0.722	0.719	0.034	21.282	<0.001
Item 10	0.354	0.353	0.055	6.440	<0.001
**SIL**					
Item 1	0.302	0.295	0.115	2.635	0.008
Item 3	0.398	0.392	0.109	3.666	<0.001
Item 5	0.320	0.313	0.122	2.619	0.009
Item 6	0.402	0.400	0.083	4.870	<0.001
Item 7	0.312	0.305	0.122	2.547	0.011
Item 9	0.813	0.808	0.035	22.971	<0.001
Item 10	0.871	0.866	0.019	45.652	<0.001
Item 12	0.675	0.670	0.047	14.396	<0.001
Item 15	0.589	0.585	0.053	11.054	<0.001
Item 16	0.569	0.565	0.067	8.465	<0.001
Item 18	0.174	0.170	0.106	1.643	0.101
Item 19	0.595	0.592	0.068	8.732	<0.001
**EPDS2**					
Item 1	0.720	0.720	0.027	26.673	<0.001
Item 2	0.469	0.470	0.042	11.264	<0.001
Item 3	0.805	0.804	0.019	43.093	<0.001
Item 4	0.689	0.688	0.034	20.054	<0.001
Item 5	0.759	0.757	0.030	25.699	<0.001
Item 6	0.473	0.471	0.056	8.481	<0.001
Item 7	0.522	0.522	0.042	12.532	<0.001
Item 8	0.745	0.744	0.025	29.254	<0.001
Item 9	0.709	0.710	0.031	23.213	<0.001
Item 10	0.183	0.186	0.074	2.468	0.014

Supplementary Table 3 Outer (factor) loadings of variables on their latent construct.
